# Identification and Somatic Characterization of the Germline *PTEN* Promoter Variant rs34149102 in a Family with Gastrointestinal and Breast Tumors

**DOI:** 10.3390/genes13040644

**Published:** 2022-04-05

**Authors:** Vittoria Disciglio, Paola Sanese, Candida Fasano, Claudio Lotesoriere, Anna Maria Valentini, Giovanna Forte, Martina Lepore Signorile, Katia De Marco, Valentina Grossi, Ivan Lolli, Filomena Cariola, Cristiano Simone

**Affiliations:** 1 Medical Genetics, National Institute of Gastroenterology—IRCCS “S. de Bellis” Research Hospital, Castellana Grotte, 70013 Bari, Italy; disciglio.labsimone@gmail.com (V.D.); sanese.labsimone@gmail.com (P.S.); fasano.labsimone@gmail.com (C.F.); forte.labsimone@gmail.com (G.F.); leporesignorile.labsimone@gmail.com (M.L.S.); demarco.labsimone@gmail.com (K.D.M.); grossi.labsimone@gmail.com (V.G.); filo.cariola@irccsdebellis.it (F.C.); 2 Oncology Unit, National Institute of Gastroenterology—IRCCS “S. de Bellis” Research Hospital, Castellana Grotte, 70013 Bari, Italy; claudio.lotesoriere@irccsdebellis.it (C.L.); ivan.lolli@irccsdebellis.it (I.L.); 3 Department of Pathology, National Institute of Gastroenterology—IRCCS “S. de Bellis” Research Hospital, Castellana Grotte, 70013 Bari, Italy; am.valentini@irccsdebellis.it; 4 Medical Genetics, Department of Biomedical Sciences and Human Oncology (DIMO), University of Bari Aldo Moro, 70124 Bari, Italy

**Keywords:** *PTEN* promoter, *PTEN* hamartoma tumor syndrome (PHTS), gastroesophageal junction adenocarcinoma, breast cancer

## Abstract

Genetic variants located in non-coding regions can affect processes that regulate protein expression, functionally contributing to human disease. Germline heterozygous mutations in the non-coding region of the *PTEN* gene have been previously identified in patients with *PTEN* hamartoma tumor syndrome (PHTS) diagnosed with breast, thyroid, and/or endometrial cancer. In this study, we report a *PTEN* promoter variant (rs34149102 A allele) that was identified by direct sequencing in an Italian family with a history of gastroesophageal junction (GEJ) adenocarcinoma and breast cancer. In order to investigate the putative functional role of the rs34149102 A allele variant, we evaluated the status of *PTEN* alterations at the somatic level. We found that PTEN protein expression was absent in the GEJ adenocarcinoma tissue of the index case. Moreover, we detected the occurrence of copy number loss involving the *PTEN* rs34149102 major C allele in tumor tissue, revealing that the second allele was somatically inactivated. This variant is located within an active regulatory region of the *PTEN* core promoter, and in silico analysis suggests that it may affect the binding of the nuclear transcription factor MAZ and hence PTEN expression. Overall, these results reveal the functional role of the *PTEN* promoter rs34149102 A allele variant in the modulation of PTEN protein expression and highlight its contribution to hereditary cancer risk.

## 1. Introduction

The rapid progress of genetic and genomic high-throughput technologies has paved the way for personalized cancer risk assessment, genetic counseling, and clinical management, underpinning the era of precision medicine [1]. However, despite these advancements, cancer genetics still poses significant challenges, including understanding the role of common and rare genomic markers in cancer susceptibility and their integration into medical practice [2].

Inherited cancer predisposition syndromes are associated with an increased risk of developing cancer. Indeed, individuals from families affected by cancer-associated syndromes with known genetic causes, such as Lynch syndrome, Peutz–Jeghers syndrome, Li–Fraumeni syndrome, familial adenomatous polyposis (FAP), *MUTYH*-associated adenomatous polyposis (MAP), *PTEN* hamartoma tumor syndrome (PHTS), and hereditary breast and ovarian cancer syndromes, are at greater risk of cancer [3,4,5]. Furthermore, previous studies have shown that cancer risk is about two-fold higher in subjects with a positive family history of cancer [6].

Several studies have identified gene polymorphisms as possible risk factors for the development of many cancers. Moreover, recent data have highlighted that common variants within genomic non-coding regions may play a role in cancer risk through the regulation of gene expression [7,8].

Advanced technologies, such as next-generation sequencing, have resulted in the identification of many genetic variants, which are often challenging to interpret. Yet, understanding their clinical significance is crucial to clarify if and how they affect disease risk and phenotypes. Moreover, characterizing rare variants in representative families may shed further light on genotype–phenotype association.

In the present study, we molecularly and clinically characterized a single-nucleotide variant (NM_000314.4:c.-1026C>A, rs34149102 A allele) located in the promoter region of the phosphatase and tensin homolog (*PTEN*) gene, which we identified in an Italian family with a history of gastroesophageal junction (GEJ) adenocarcinoma and breast cancer.

## 2. Materials and Methods

### 2.1. Patient Recruitment

The index patient and his relatives underwent molecular genetic testing as part of the routine clinical diagnostic assessment performed at our institute. Written informed consents to perform testing and further studies on tissue samples was obtained from the patient and his relatives using a form approved by the competent ethics committee, in line with the principles of the Declaration of Helsinki and any other applicable local ethical and legal requirements (protocol code N_ 170, date of approval 31 October 2016).

### 2.2. DNA Extraction and Sanger Sequencing

Genomic DNA was extracted from peripheral blood with the QIAamp DNA Blood Mini Kit (Qiagen, Carlsbad, CA, USA) according to the manufacturer’s instructions. The promoter region and complete coding region of the *PTEN* gene were screened for mutations using primer sequences previously published by Tan et al. [9]. PCR sequencing and capillary electrophoresis were performed on an Applied Biosystems 3130 Genetic Analyzer (Thermo Fisher Scientific, Waltham, MA, USA). Mutations and polymorphisms were confirmed in independently amplified PCR products. The global population frequency of the identified *PTEN* variant was retrieved from the 1000 Genome [10] and gnomAD [11] databases.

### 2.3. Next-Generation Sequencing

The Ion AmpliSeq Custom Panel (BRCA Reflex), comprising 25 genes involved in major hereditary cancer predisposition syndromes (*APC, ATM, BARD1, BMPR1A, BRIP1, CDH1, CDK4, CDKN2A, CHEK2, EPCAM, MLH1, MRE11A, MSH2, MSH6, MUTYH, NBN, PALB2, PMS2, PTEN, RAD50, RAD51C, RAD51D, SMAD4, STK11, TP53*), was used for targeted sequencing. The library was generated from 10 ng of the whole-blood DNA of the index patient using the Ion AmpliSeq Chef Solutions DL8 Kit (Thermo Fisher Scientific, Waltham, MA, USA) on an Ion Chef System (Thermo Fisher Scientific, Waltham, MA, USA). Two premixed pools of primer pairs were used to generate the sequencing libraries. The clonal amplification and chip loading of the libraries were performed on the Ion Chef System. Subsequently, the prepared libraries were sequenced on an Ion GeneStudio S5 Prime System (Thermo Fisher Scientific, Waltham, MA, USA) using the Ion 510™ & Ion 520™ & Ion 530™ Kit and the Ion 520 Chip Kit (Thermo Fisher Scientific, Waltham, MA, USA) according to the manufacturer’s instructions. Data analysis was performed using the Torrent Suite Software v.5.12.1 (Thermo Fisher Scientific, Waltham, MA, USA). Reads were aligned to the hg19 human reference genome. The mean average read depth and the percentage of reads that mapped to the region of interest (ROI) out of the total number of reads (reads on target) were calculated using the Coverage Analysis plugin (Torrent Suite v.5.12.1 software, Thermo Fisher Scientific, Waltham, MA, USA). For each sample, the percentage of the ROI with a minimum coverage of 20X was calculated using the amplicon coverage matrix file.

### 2.4. Sample Collection and Immunohistochemical (IHC) Analysis

GEJ adenocarcinoma tissue was surgically removed at the National Institute for Gastroenterology, IRCCS “S. de Bellis” Research Hospital, Castellana Grotte (BA), Italy. For histological examination, tissue samples were routinely fixed with formalin and embedded in paraffin at the Pathology Department. Sections stained with hematoxylin and eosin were reviewed by a pathologist to confirm the adequacy of the sample and to evaluate the pathological characteristics of the tumor. For IHC detection, 4 µm sections were cut and mounted on Apex Bond IHC slides (Leica Biosystems, Buffalo Grove, IL, USA). IHC staining procedures were performed on the BOND III automated immunostainer (Leica Biosystems, Buffalo Grove, IL, USA) from dewaxing to counterstaining with hematoxylin. Tissue sections were incubated with an anti-PTEN primary antibody (rabbit mAb 138G6, Cell Signaling Technology, Danvers, MA, USA; dilution 1:200) for 30 min at room temperature. Sections were retrieved in Epitope Retrieval Solution 2 (High pH) (Leica Biosystems, Buffalo Grove, IL, USA). The Bond Polymer Refine Detection Kit (Leica Biosystems, Buffalo Grove, IL, USA) was used as a visualization and chromogen reagent according to the manufacturer’s instructions. PTEN immunoreactivity was defined by the presence of at least 5% positive cells with cytoplasmic and/or nuclear staining. Staining in normal cells and stroma was used as a positive control.

### 2.5. Second-Hit Somatic Alteration Analysis

Methylation Specific Multiplex Ligation Dependent Probe Amplification (MS-MLPA, MRC Holland, Amsterdam, The Netherlands) analysis was performed using formalin-fixed, paraffin-embedded (FFPE), archival GEJ adenocarcinoma and paired with normal tissue specimens from the index case. Five 5 μm sections of GEJ adenocarcinoma and paired normal tissues were obtained from each paraffin-embedded block. Macrodissection was carried out on hematoxylin-eosin-stained sections, and only tissue representative of tumoral areas was used for DNA extraction. Genomic DNA was purified using the GeneRead DNA FFPE Kit (Qiagen, Carlsbad, CA, USA) according to the manufacturer’s instructions.

To perform methylation-specific MS-MLPA, we used the ME001-D1 Tumor Suppressor-1 Kit (MRC Holland, Amsterdam, The Netherlands). This kit enables a total of 25 tumor suppressor genes (*APC*, *ATM*, *BRCA1*, *BRCA2*, *CADM1*, *CASP8*, *CD44*, *CDH13*, *CDKN1B*, *CDKN2A*, *CDKN2B*, *CHFR*, *DAPK1*, *ESR1*, *FHIT*, *GSTP1*, *HIC1*, *KLLN*, *MLH1*, *PTEN*, *RARB*, *RASSF1*, *TIMP3*, *TP73*, *VHL*) to be analyzed for aberrant promoter methylation and copy number variations. Experimental procedures were carried out according to the manufacturer’s instructions. Briefly, a total of 100 ng of DNA was denatured and then annealed overnight at 60 °C to the MS-MLPA probe mix. Subsequently, half of the sample was ligated by adding Ligase-65 (MRC-Holland, Amsterdam, The Netherlands) and incubated at 49 °C for 30 min, whereas the other half was digested using the Hha1 restriction enzyme. Ligation products were amplified by PCR according to the manufacturer’s instructions (MRC-Holland, Amsterdam, The Netherlands). The ligase enzyme was inactivated by heating at 98 °C for 5 min. PCR was performed according to the manufacturer’s instructions (MRC-Holland, Amsterdam, The Netherlands). Subsequently, PCR reaction fragments were separated and visualized on an automated sequencer (Applied Biosystems 3130 Genetic Analyzer, Thermo Fisher Scientific, Waltham, MA, USA). A normal DNA sample was used as a control.

Promoter methylation and copy number variations were analyzed using Coffalyser software (MRC-Holland, Amsterdam, The Netherlands). Aberrant methylation was scored when the calculated methylation ratio was >25%. Any methylation percentage below this level was considered as background. As previously reported, ratios were interpreted as mild hypermethylation (25–50%), moderate hypermethylation (50–75%), or extensive hypermethylation (>75%) [12]. Copy number analysis was performed on MLPA results from undigested samples using Coffalyser software algorithms to compute the copy number ratio. Thresholds to detect copy number gains and losses were set to 1.3 and 0.7, respectively. The mutational status of the *PTEN* promoter in GEJ adenocarcinoma and normal (peripheral blood and GEJ normal tissue) samples from the index case was screened using PCR and direct Sanger sequencing. PCR was carried out in a 50 µL volume containing 10 ng of DNA and 0.5 µM of each primer (For: 5′-TCAGTAGAGCCTGCGGCTTG-3′; Rev: 5′-TCCCCTCGGTCTTCCGAG-3′) using the Q5 High Fidelity DNA Polymerase (New England Biolabs, MA, USA) according to the manufacturer’s instructions.

### 2.6. In Silico Analysis

Three transcription factor (TF) prediction tools (i.e., MatInspector, PROMO, and OncoBase [13,14,15]) were used to search for potential TF binding sites in the region of the identified *PTEN* promoter variant. This analysis was carried out by inputting a 40 bp sequence encompassing the rs34149102 genomic position (−1043/−1003) to search for binding sites comprising the C or A allele.

The public database RegulomeDB [16] includes high-throughput experimental datasets from the Encyclopedia of DNA Elements (ENCODE) project, Gene Expression Omnibus, and other sources. We searched this database for rs34149102 and analyzed the rank and ChIP-seq data, filtering “cancer cells” and “epithelial cells” as cell types, and then choosing “MCF-7”.

## 3. Results

### 3.1. Medical History and Genetic Findings 

**Figure 1:**
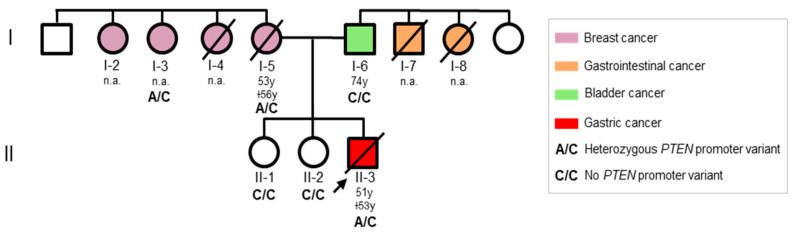
Pedigree of the family involved in this study. Squares indicate men, circles represent women. The arrow indicates the index patient. Unfilled symbols indicate unaffected individuals. Slashed symbols denote dead individuals. The following information is provided below each filled symbol: age at diagnosis (y = years; n.a. = not available), age at death (†).

In the current study, we examined an Italian family with a medical history of GEJ adenocarcinoma and breast cancer. A 51-year-old man, who previously developed several lipomas (*n* = 19), was diagnosed with differentiated adenocarcinoma in fragments of the gastroesophageal junction mucosa (Figure 1, II-3). The patient underwent an Ivor-Lewis gastroesophagectomy due to the presence of an eccentric and ulcerated cardial neoformation infiltrating the esophagus and nearby lymph nodes, which were also resected by lymphadenectomy. Histological examination confirmed the previous diagnosis and revealed perineural, celiac, and periesophageal tripod lymph node infiltration. Later on, the patient completed one cycle of EOX (epirubicin, oxaliplatin, and capecitabine) chemotherapy followed by two cycles of XELOX (capecitabine and oxaliplatin). A subsequent computed tomography scan revealed no tumoral recurrence and enlarged nodal lesions. One year later, the patient developed a lipoma (6.3 cm × 2.8 cm) on the volar side of the elbow and two metastatic lesions originating from the GEJ adenocarcinoma, one in the right biceps muscle (3.4 cm × 2.7 cm × 5.3 cm) and the other in the adjacent vascular-nerve bundle (1.5 cm × 1.3 cm × 4.4 cm). Subsequently, the patient underwent disarticulation of the right upper limb for recurrent metastasis (squamous cell carcinoma exulcerating the epidermis and extensively involving the dermis and hypodermis layers, muscle, and bone tissue) originating from the GEJ adenocarcinoma. During a physical examination, macrocephaly and multiple oral papillomas on the tongue were documented. Based on the presence of multiple and meta-synchronous tumors, the patient was referred to genetic counseling but died shortly afterward at 53 years of age. 

**Figure 2:**
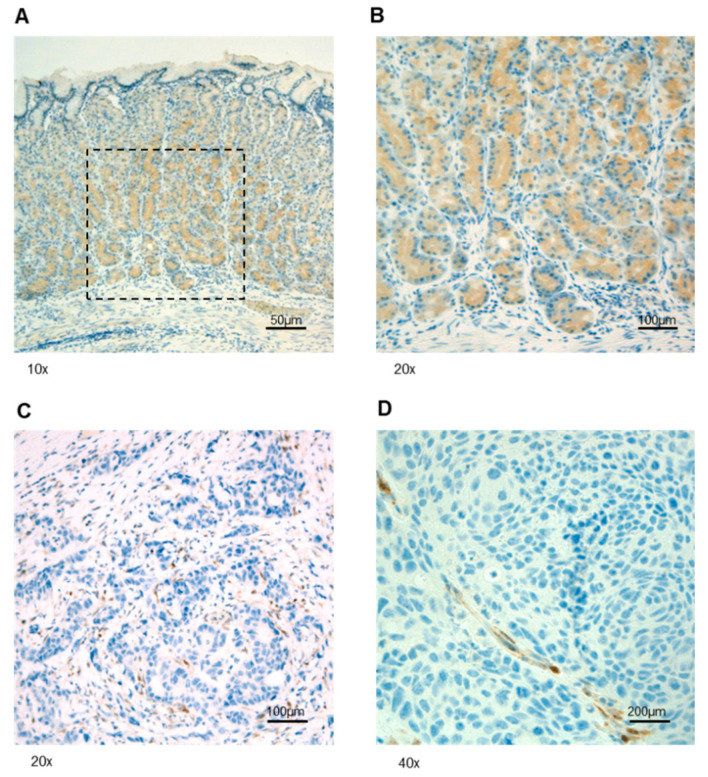
Representative IHC staining of PTEN protein expression in tissues from the index case. (**A**) Normal gastric epithelium showing cytoplasmic staining. (**B**) Higher magnification of the squared area shown in (**A**). (**C**,**D**) GEJ tissue showing positive staining in normal stromal (**C**) and endothelial (**D**) cells and a lack of reactivity in cancer cells.

**Figure 3:**
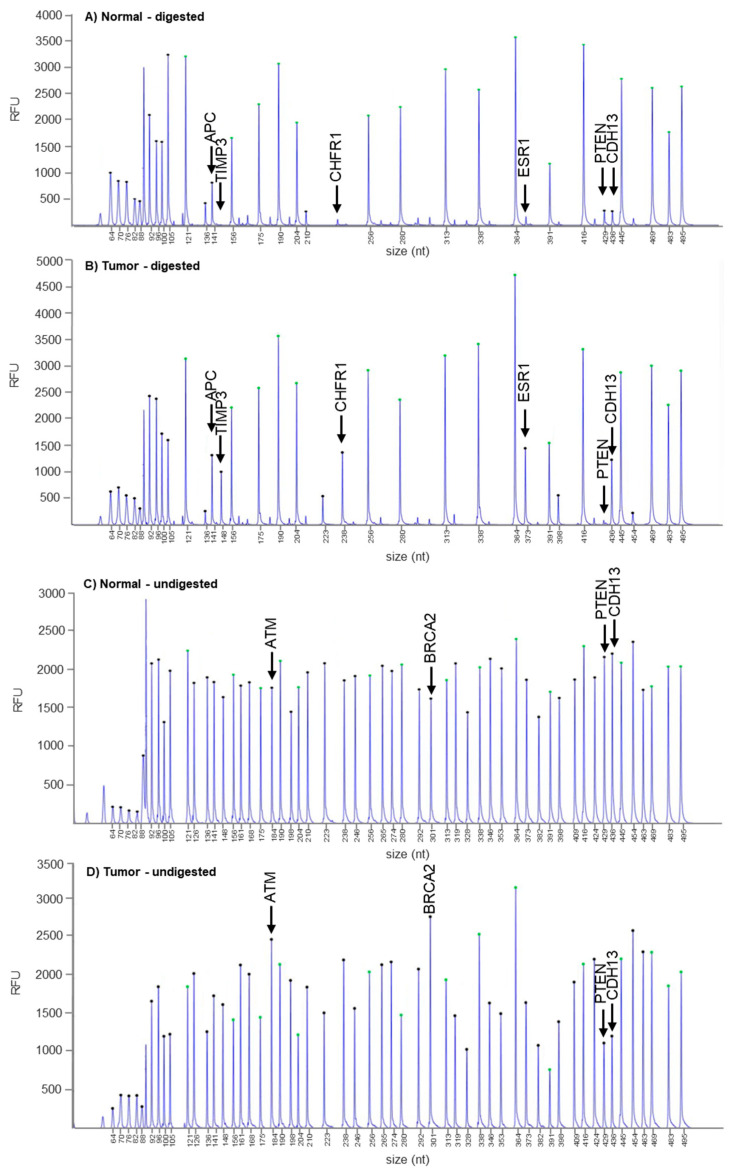
Detection of methylation and aberrant copy number variations in tumor compared to normal tissue from the index patient. (**A**,**B**) MS-MLPA assay with HhaI enzyme treatment for methylation analysis in normal (**A**) and tumor (**B**) GEJ tissue from the index patient showing *APC, ESR1, CHFR1, CDH13*, and *TIMP3* hypermethylation (methylation ratio 40–65%) in tumor tissue. (**C**,**D**) MS-MLPA assay without HhaI enzyme treatment for copy number analysis in normal (**C**) and tumor (**D**) GEJ tissue from the index patient showing *PTEN* and *CDH13* losses (copy number ratio: 0.5 and 0.63, respectively) and *ATM* and *BRCA2* gains (copy number ratio: 1.43 and 1.58, respectively) in tumor tissue.

**Figure 4:**
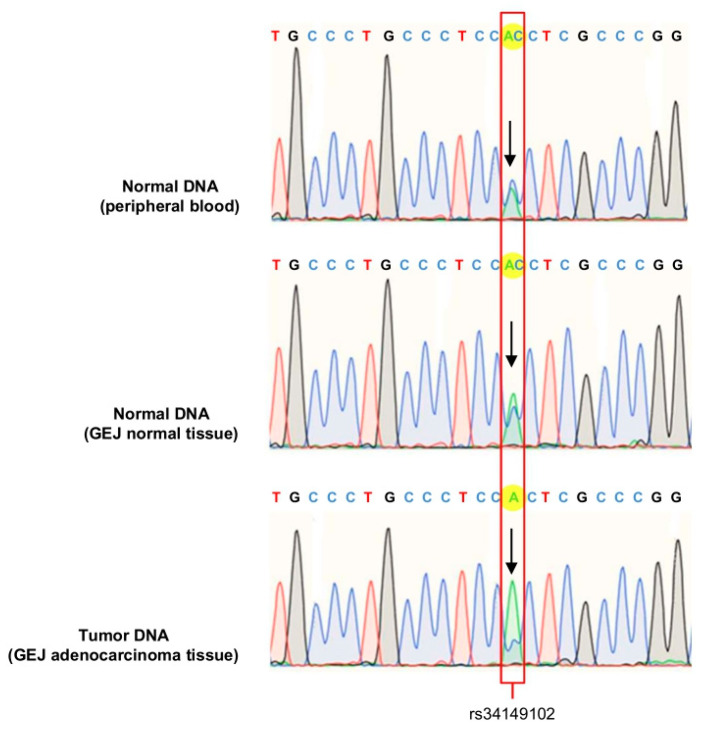
Sanger sequencing electropherograms of the region around the *PTEN* rs34149102 locus in normal DNA (peripheral blood and GEJ normal tissue) and tumor DNA (GEJ adenocarcinoma tissue), showing the loss of the *PTEN* rs34149102 major C allele in tumor tissue.

**Figure 5:**
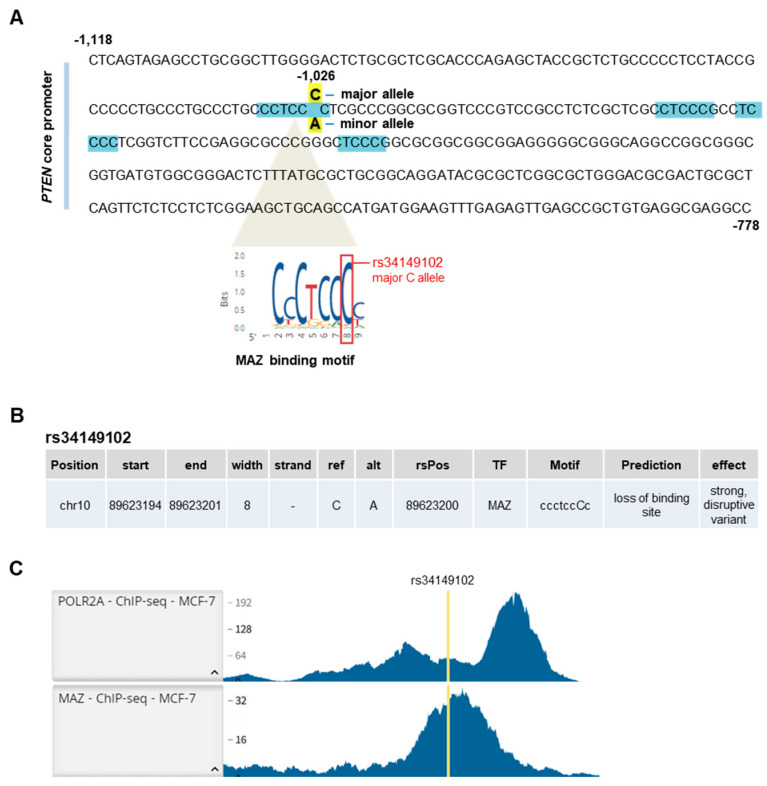
*In silico* analysis of the region encompassing the rs34149102 locus. (**A**) *PTEN* core promoter region (bp −1118/−778). The MAZ recognition motif encompassing the rs34149102 major C allele (bp −1026) and the other three putative MAZ binding sites are highlighted in light blue. (**B**) Predicted effects of the rs34149102 A allele on MAZ binding by using MatInspector, PROMO, and OncoBase prediction tools. ref = reference allele; alt = alternative allele; rsPos = rs34149102 genomic position. (**C**) RegulomeDB ChIP-seq data of Pol II (POLR2A) and MAZ binding in the promoter region encompassing the rs34149102 locus in the MCF-7 breast cancer cell line.

As summarized in Figure 1, the index case had a positive family history of tumors of the gastrointestinal tract and breast cancer. His father developed bladder cancer at 74 years of age (Figure 1, I-6), and one paternal uncle and one paternal aunt died from tumors of the gastrointestinal tract (no clinical documentation available) (Figure 1, I-7, I-8). His mother developed breast cancer at 53 years of age and died three years later (Figure 1, I-5). Three maternal aunts were diagnosed with breast cancer, one of whom died from the disease (Figure 1, I-2, I-3, I-4). 

Based on clinical findings (macrocephaly, multiple oral papillomas, and lipomas) and on the positive family history of gastrointestinal and breast cancer, we performed a molecular study on the *PTEN* gene of the index patient. This analysis identified a heterozygous single-nucleotide variant (NM_000314.4:c.-1026C>A, rs34149102) located in the *PTEN* promoter on chromosome 10 (human genomic localization chr10:89623200C>A, GRCh37). To assess the frequency of the identified *PTEN* promoter variant in the general population, we queried major population databases, including 1000 Genome and gnomAD. This analysis showed that the rs34149102 A allele variant is found in less than 1% of the worldwide population.

In order to assess the potential role of additional genetic factors in the medical and family history of the index patient, we analyzed, by next-generation sequencing, a panel of genes primarily involved in hereditary cancer predisposition syndromes (*APC, ATM, BARD1, BMPR1A, BRIP1, CDH1, CDK4, CDKN2A, CHEK2, EPCAM, MLH1, MRE11A, MSH2, MSH6, MUTYH, NBN, PALB2, PMS2, PTEN, RAD50, RAD51C, RAD51D, SMAD4, STK11, TP53*). This analysis did not identify potential causal variants in the coding regions of these genes that could contribute to the medical history of the index patient and his family.

To better elucidate the role of the identified *PTEN* promoter variant in the clinical phenotypes of the family under study, we performed a segregation analysis. This variant was not detected in the healthy first-degree relatives tested (Figure 1, II-1, II-2), nor in the father of the index patient (Figure 1, I-6), but was found in the mother (Figure 1, I-5) and in one maternal aunt (Figure 1, I-3). In this family, the heterozygous *PTEN* variant NM_000314.4:c.-1026C>A (rs34149102 A allele) seems to be associated with the clinical findings of the index patient (macrocephaly, multiple oral papillomas, lipomas, and GEJ adenocarcinoma) and his relatives (breast cancer), which are, in part, included in the phenotypic spectrum of PHTS [17,18].

### 3.2. PTEN Somatic Alterations in GEJ Adenocarcinoma Samples

In order to characterize the rs34149102 *PTEN* promoter variant at the somatic level, we performed IHC staining to analyze PTEN protein localization in normal and neoplastic tissue from the index patient. PTEN protein expression was detected in normal GEJ epithelial, stromal, and endothelial cells. In contrast, GEJ neoplastic cells showed a loss of PTEN protein expression. Representative immunostainings are reported in Figure 2. These findings suggest that the *PTEN* rs34149102 A allele variant may affect PTEN protein expression.

Subsequently, we sought to ascertain whether the complete absence of PTEN protein expression in the index patient’s cancer cells could be due to the aberrant methylation of cytosine–phosphate–guanine (CpG) sites located in the *PTEN* promoter region. To this end, we evaluated epigenetic changes by using the MS-MLPA probe set ME001-D1 (MRC Holland) in formalin-fixed, paraffin-embedded (FFPE) GEJ adenocarcinoma tissue samples from the index patient and then compared the results with those gathered in corresponding normal samples. A total of 25 known oncosuppressor genes were analyzed for aberrant methylation (Figure 3). MS-MLPA analysis was performed in duplicate and generated reproducible ratios (data not shown). Our results revealed no *PTEN* promoter hypermethylation; however, promoter hypermethylation events were identified in 5/25 genes, i.e., *APC*, *ESR1*, *CHFR1*, *CDH13*, and *TIMP3* (Figure 3A,B). Notably, most of these genes are known to be potential diagnostic biomarkers for gastrointestinal and breast cancer [19,20,21,22,23]. 

In order to better characterize the status of both *PTEN* alleles, we then analyzed by MS-MLPA the same 25 genes for copy number gains/losses in normal and tumor samples from the index patient. Based on the DNA isolated from 20 normal gastrointestinal samples, we set the thresholds for gains and losses to 1.3 and 0.7, respectively. Overall, we detected copy number gains in *ATM* and *BRCA2* and copy number losses in *PTEN* and *CDH13* (Figure 3C,D). Altogether, these findings suggest that the complete loss of PTEN expression could be due to the occurrence of a two-hit event, i.e., the presence of the identified rs34149102 A allele variant in the first allele and the deletion of the second allele.

To gain further insight into the occurrence of the somatic inactivation of the second allele, we analyzed GEJ adenocarcinoma and normal tissue from the index patient to evaluate the mutational status of the *PTEN* promoter. The *PTEN* sequence electropherogram of normal DNA (GEJ normal tissue and peripheral blood samples) revealed equivalent proportions of the rs34149102 major C allele and rs34149102 minor A allele. In contrast, the rs34149102 minor A allele was predominant in DNA from tumor tissue, indicating the occurrence of copy number loss involving the *PTEN* rs34149102 major C allele in the index patient’s GEJ adenocarcinoma tissue (Figure 4).

### 3.3. In Silico Analysis

Previous works suggested that *PTEN* promoter variants can be relevant to PHTS phenotypes by affecting transcriptional and translational processes that regulate PTEN expression [24,25,26]. Interestingly, the *PTEN* promoter rs34149102 A allele variant has been shown to correlate with the decreased expression of full-length *PTEN* transcript in PHTS patients [27]. 

The rs34149102 locus is located 1026 bp upstream of the *PTEN* transcription start site (TSS), within its core promoter region, which lies between bp −1118 and −778 relative to the TSS and was shown to have optimal promoter activity [28]. The core promoter facilitates the assembly of the transcription machinery, including RNA polymerase II (Pol II) [29]. In certain circumstances, Pol II transcription can be activated in a tissue-specific manner by regulatory elements [30], which are genomic regions that regulate differentially expressed genes. The localization of rs34149102 in the core promoter prompted us to ascertain whether it could affect the regulation of PTEN protein expression. The core promoter region comprises binding sites for several TFs, including p53, NF-κB, Ap2, MAZ, Sp1, and E4F [28]. In particular, while searching for consensus motifs for these TFs, we found four putative MAZ binding sites located in the core promoter region. Among these, only one perfectly matches the entire consensus motif, and it encompasses the rs34149102 locus (Figure 5A). 

To evaluate the potential functional significance of the C to A change in the rs34149102 locus (human genomic localization chr10:89623200C>A, GRCh37), we performed an *in silico* analysis to predict whether it may affect TF binding. Three different TF prediction tools (i.e., MatInspector, PROMO, and OncoBase) revealed that the presence of the minor A allele may indeed alter MAZ binding to the *PTEN* promoter in the region encompassing rs34149102 by potentially altering its binding site (Figure 5B). These findings led us to speculate that the rs34149102 locus may have transcription regulatory properties and that the presence of the rs34149102 A allele may affect MAZ binding to the *PTEN* promoter, thereby influencing *PTEN* gene expression. 

We then used the RegulomeDB database to gather further evidence on the putative functional role of rs34149102. The rs34149102 genomic position was ranked with a 2a classification, meaning that it is likely to influence TF binding at matched motifs, DNase footprint, and DNase peak [31]. We searched the RegulomeDB database for ChIP-seq data of gastroesophageal and breast samples, i.e., the tissues affected by tumors in the family of the index case. However, no gastroesophageal sample was reported in the database. On the other hand, data from the MCF-7 human breast cancer cell line showed MAZ occupancy in the region surrounding rs34149102, which is significantly enriched for Pol II binding (Figure 5C). Overall, this evidence further suggests that rs34149102 is located in an active regulatory region of the core promoter regulating *PTEN* transcription and that the presence of the minor A allele may influence its expression due to the loss of a MAZ recognition motif.

## 4. Discussion

Genetic variants located in non-coding regions may regulate the expression of target genes by altering TF binding motifs, epigenetic modifications, chromatin accessibility, or 3D genome conformation, thereby functionally contributing to genetic disorders and tumorigenesis [7,8,32]. Indeed, genome-wide association studies have revealed the potential role of these variants in susceptibility to complex diseases, such as breast cancer and GEJ adenocarcinoma [33,34]. An increasing number of variants have been identified in the non-coding regions—such as promoters, introns, and untranslated regions (UTRs)—of cancer susceptibility genes through the germline clinical testing of cancer cases by high-throughput genomic technologies [35].

In this study, we report a *PTEN* gene promoter variant (NM_000314.4:c.-1026C>A, rs34149102) that was identified by direct sequencing in an Italian family with a history of GEJ and breast cancer.

The *PTEN* gene is located at 10q23.3 and plays a key role as a tumor suppressor in human cancers. It encodes a dual-specificity lipid and protein phosphatase that antagonizes the phosphoinositol-3-kinase (PI3K)/AKT signaling pathway [36,37]. Previous studies have shown that *PTEN* expression can be regulated at the transcriptional level and that subtle variations in PTEN dosage may result in increased cancer susceptibility and be associated with cellular proliferation and tumorigenesis [38,39].

*PTEN* transcriptional regulation is mediated by a variety of TFs, which bind to the promoter region of the gene at specific times and in different cell types [40]. Moreover, the epigenetic regulation of the *PTEN* promoter by hypermethylation has been observed in breast, colorectal, and gastric cancers [41,42,43]. Germline mutations involving the *PTEN* gene have been associated with a spectrum of related genetic disorders collectively referred to as *PTEN* hamartoma tumor syndromes (PHTS), which include Cowden syndrome (CS), Bannayan–Riley–Ruvalcaba syndrome (BRRS), adult Lhermitte–Duclos disease, and autism spectrum disorders associated with macrocephaly [44]. To date, however, strong evidence suggesting a correlation between specific *PTEN* mutations and clinical presentations has not been provided.

The clinical features of PHTS patients with *PTEN* gene mutations are extensively variable, showing age-related penetrance, even between patients with the same mutation and patients in the same family. Specifically, the clinical spectrum of PHTS includes several manifestations characterized by developmental delay, complex and multifaced overgrowth phenotypes (e.g., macrocephaly), gastrointestinal hamartomas, the glycogenic acanthosis of the esophagus, vascular malformations, lipomas, hemangiomas, and skin abnormalities [45]. Additionally, increased risk for several tumor types, including breast, endometrial, and thyroid cancer, has been reported in patients with PHTS, while the risk of other tumors (e.g., colorectal and renal cancer*)* has not been accurately defined yet [44]. To date, esophageal and gastric cancers have rarely been reported in PHTS patients and are not considered part of the PHTS spectrum [46,47,48,49].

The clinical features detected in the index case of the present study included major (macrocephaly, multiple oral papillomas on the tongue) and minor (lipomas) clinical criteria of PHTS. Moreover, the mother and three maternal aunts of the index case had a history of breast cancer, one of the major criteria for the clinical diagnosis of PHTS. Thus, the clinical findings of the index case and his relatives led to a tentative diagnosis of PHTS.

In order to gain more insight into the potential genetic causes underlying the clinical phenotypes observed in this family, we performed a molecular analysis of the coding and promoter regions of the *PTEN* gene in the index case. This analysis did not reveal loss-of-function mutations in the *PTEN* coding region or large deletions involving this gene. However, we identified a variant (rs34149102 A allele) located within the *PTEN* core promoter region. A segregation analysis showed that this variant was inherited from the mother and was also present in one maternal aunt with breast cancer. No pathogenic variant was identified in the index case by next-generation sequencing in other major genes involved in hereditary predisposition syndromes.

The frequency of the identified variant was assessed by querying various population databases, including 1000 Genome and gnomAD. This analysis revealed that the rs34149102 A allele is a low-frequency variant since it is found in less than 1% of the worldwide population.

To date, germline heterozygous mutations at the single nucleotide level within the promoter of the *PTEN* gene have been identified in patients with CS and/or BRRS diagnosed with breast, thyroid, and/or endometrial cancer. In CS patients with *PTEN* promoter mutations, protein analysis showed that these mutations were associated with aberrant PTEN expression [24,25,26]. Moreover, germline heterozygous deletions involving a highly conserved region upstream of the *PTEN* regulatory elements located in the *PTEN* core promoter have been identified in patients with CS [26,50]. Altogether, these findings suggest that alterations in the *PTEN* promoter region and/or regulatory elements can contribute to disease pathogenesis.

Importantly, Sarquis et al. detected the *PTEN* promoter rs34149102 A allele variant in a heterozygous state in patients (*n* = 7) with CS/CS-like phenotypes. In this study, the authors found significantly reduced *PTEN* full-length transcript levels in these patients compared with controls (*n* = 27) not harboring genetic variants involving the *PTEN* gene [27]. In an additional study investigating the potential effect of *PTEN* promoter variants on tumor characteristics and disease outcomes in 2412 breast cancer patients, an association was reported between the *PTEN* promoter rs34149102 A allele variant and a worse prognosis (worse long-term survival and the increased proliferation of tumor cells) [51].

Overall, this evidence supports the hypothesis that the *PTEN* promoter rs34149102 A allele variant is implicated in the modulation of *PTEN* inactivation at the transcriptional level, thereby influencing PHTS specific phenotypes.

In order to characterize the role of this variant in the index case’s tumor, we evaluated PTEN protein expression in GEJ adenocarcinoma samples by IHC staining. This analysis revealed a lack of PTEN immunoreactivity in cancer cells, suggesting that the presence of the identified *PTEN* promoter variant could affect PTEN protein expression. The complete absence of PTEN expression in cancer cells prompted us to investigate the occurrence of a second-hit mutation event in the GEJ tumor tissue of the index case.

The methylation status and copy number variations of the *PTEN* gene were investigated in GEJ adenocarcinoma samples from the index case. This analysis excluded the alterations of *PTEN* methylation status and revealed the presence of a deletion involving one copy of the *PTEN* gene. Additionally, direct sequencing indicated that the complete inactivation of the *PTEN* gene was associated with the somatic loss of the rs34149102 major C allele in the GEJ adenocarcinoma tissue of the index case. The occurrence of this second-hit mutation event and the loss of PTEN protein expression support the functional impact of the rs34149102 A allele on GEJ adenocarcinoma pathogenesis in the index case.

The *PTEN* variant identified in our family lies in the *PTEN* core promoter region (−1118 to −778 bp upstream of the TSS) [28]. A bioinformatics prediction analysis showed that the *PTEN* core promoter region is enriched in binding sites for several nuclear TFs (e.g., p53, NF-κB, Ap2, MAZ, Sp1, E4F) [28] and that the rs34149102 C allele is part of a specific consensus motif for MAZ that may be altered by the variant. ChIP-seq data confirmed an enrichment in MAZ recruitment in the region surrounding rs34149102 in the MCF-7 breast cancer cell line. These results led us to speculate that MAZ may play a role in the regulation of *PTEN* expression in cancer cells. MAZ is a six-zinc finger protein with a G-rich binding motif, which acts as a TF and is ubiquitously expressed in most tissues. Previous studies have shown that MAZ activates the expression of a variety of genes, including c-Myc, insulin, VEGF, and the RAS gene family in cancer cells [52,53,54]. Moreover, prior studies have shown that deregulated expression of MAZ is closely related to the progression of various tumors, such as breast cancer, liposarcoma, prostate cancer, and glioblastoma [52,55,56]. Intriguingly, MAZ has been described as a negative regulator of AKT signaling [57]. It is therefore tempting to speculate that the rs34149102 A allele may alter MAZ binding, leading to decreased *PTEN* expression and the activation of AKT signaling. This would support the potential pathogenic relevance of the rs34149102 A allele.

Taken together, these observations indicate that the rs34149102 A allele variant may induce *PTEN* promoter dysregulation and thus affect processes that govern PTEN protein expression. *PTEN* promoter alterations identified in PHTS cases have been associated with aberrant PTEN protein expression [24,25,26], suggesting that *PTEN* promoter variants may affect the transcriptional and/or translational regulation of PTEN protein expression, thereby contributing to PHTS pathogenicity.

Despite substantial *in silico* evidence, a limitation of our study is the lack of experimental validation of the functional impact of the rs34149102 A variant on MAZ binding to the *PTEN* core promoter region. In this light, additional functional studies are needed to address the potential of the rs34149102 C to A variation to impair MAZ binding efficiency and its effect on PTEN expression as a molecular mechanism underlying the involvement of the rs34149102 A allele variant in PHTS syndromes.

## Data Availability

Not applicable.
